# Tripterygium glycosides improve abnormal lipid deposition in nephrotic syndrome rat models

**DOI:** 10.1080/0886022X.2023.2182617

**Published:** 2023-03-06

**Authors:** Bidan Zheng, Dongfang Lu, Xiuping Chen, Yinghua Yin, Weiying Chen, Xiaowan Wang, Huanmei Lin, Peng Xu, Aihua Wu, Bo Liu

**Affiliations:** a Guangdong Provincial Key Laboratory of Clinical Research on Traditional Chinese Medicine Syndrome, The Second Clinical Medical College, Guangzhou University of Chinese Medicine, Guangzhou, China; b Guangzhou Key Laboratory of Chirality Research on Active Components of Traditional Chinese Medicine, Guangzhou, China; c State Key Laboratory of Dampness Syndrome of Chinese Medicine, Guangzhou, China; d Guangdong Provincial Key Laboratory of Chinese Medicine for Prevention and Treatment of Refractory Chronic Diseases, The Second Clinical Medical College, Guangzhou University of Chinese Medicine, Guangzhou, China

**Keywords:** Doxorubicin, nephrotic syndrome, lipotoxicity, tripterygium glycosides

## Abstract

**Objective:**

The purpose of this study was to determine the effect of tripterygium glycosides (TGs) on regulating abnormal lipid deposition in nephrotic syndrome (NS) rats.

**Methods:**

Sprague-Dawley (SD) rats were injected with 6 mg/kg doxorubicin to construct nephrotic syndrome models (*n* = 6 per group), and then administered with TGs (10 mg/kg·d^−1^), prednisone (6.3 mg/kg·d^−1^), or pure water for 5 weeks. Biomedical indexes, such as urine protein/creatinine ratio (PCR), blood urea nitrogen (BUN), serum creatinine (Scr), serum albumin (SA), triglycerides (TG), total cholesterol (TC)were investigated to evaluate the renal injury of rats. H&E staining experiment was used to assess the pathological alterations. Oil Red O staining was used to assess the level of renal lipid deposition. Malondialdehyde (MDA) and glutathione (GSH) were measured to assess the extent of oxidative damage to the kidney. TUNEL staining was used to assess the status of apoptosis in the kidney. Western blot analysis was performed to examine the levels of relevant intracellular signaling molecules.

**Results:**

After treatment with TGs, those tested biomedical indexes were significantly improved, and the extent of kidney tissue pathological changes and lipid deposition in the kidney was diminished. Treatment with TGs decreased renal oxidative damage and apoptosis. Regarding the molecular mechanism, TGs significantly increased the protein expression levels of Bcl-2 but decreased the levels of CD36, ADFP, Bax, and Cleaved caspase-3.

**Conclusion:**

TGs alleviates renal injury and lipid deposition induced by doxorubicin, suggesting that it may be a new strategy for reducing renal lipotoxicity in NS.

## Graphical Abstract

**Figure:**
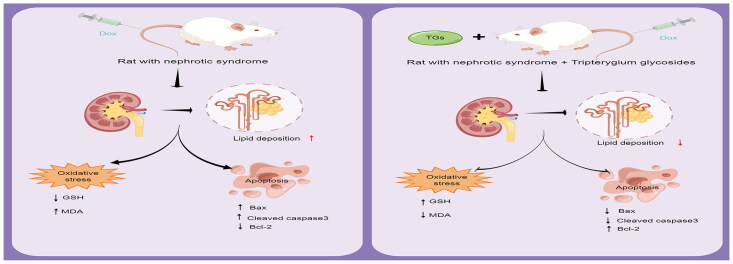

## 1. Introduction

Lipotoxicity is marked by an unusual excess accumulation of lipids in non-adipose tissues, which causes deleterious effects [1]. The kidney is one of the main target organs for lipotoxicity damage [2]. Renal parenchymal cell lipid deposition causes cellular dysfunction and apoptosis [3]. Doxorubicin-induced nephrosis in rats is a representative animal model of nephrotic syndrome [4]. Doxorubicin induces nephrotoxicity *via* renal oxidative stress, inflammation, and apoptosis [5]. In addition, doxorubicin disrupts lipid metabolism, causing the development of lipotoxicity [6], which aggravates kidney damage.

TGs is multicomponent extracts from the Chinese herb, *Tripterygium wilfordii* (thunder god vine), which exhibit excellent immunosuppressive and anti-inflammatory activities, and it shows satisfactory efficacy in nephrotic syndrome [7]. Studies have suggested that TGs plays an important role in regulating lipid metabolism. TGs effectively improves the function of impaired kidneys by promoting triglycerides (TG) catabolism *via* modulation of adipose triglyceride lipase [8]. The two main active components of TGs, triptolide, and celastrol, could reduce excessive lipid accumulation [9,10]. However, it remains unknown whether TGs have a favorable therapeutic effect on lipotoxicity caused by NS. Thus, the present study performed a series of experiments to determine the effects of TGs on NS-induced lipotoxicity.

## 2. Methods

### 2.1. Animals

Eight-week-old SPF adult male Sprague–Dawley rats (200 ± 20 g) were purchased from Guangdong Medical Laboratory Animal Center (Guangzhou, China) and maintained under specific pathogen-free conditions, 20 ± 2 °C temperature, 50 ± 10% humidity, regular 12-h dark/light cycles, and the rats were allowed free access to food and water. After a week of acclimatization, the rats were randomly divided into the following four groups (*n* = 5–6 per group): control group, model group, TGs group, and prednisone group. All *in vivo* experiments were performed according to protocols approved by the Animal Care and Use Committee of Guangdong Provincial Hospital of Traditional Chinese Medicine.

### 2.2. Experimental design

All rats (except the control group) were given 6 mg/kg DOX *via* a single tail vein injection to construct a nephrotic syndrome model. After 3 weeks, the rats were regrouped according to the PCR and treated as follows: the TGs group was treated with 10 mg/kg·d^−1^ TGs (i.g.); the prednisone group was treated with 6.3 mg/kg·d^−1^ prednisone (i.g.); and the model and control groups were given equal amounts of distilled water. The body weight was recorded once a week. Rat urine was collected to detect urinary protein and creatinine on the 5th week of dosing. After 5 weeks of continuous dosing, all rats were euthanized with sodium pentobarbital. Blood was collected, and serum was separated by centrifugation at 3500 × g for 15 min. Kidneys were removed and stored according to the experimental needs.

### 2.3. Kidney index

All animals were weighed before being euthanized. Kidney tissues were collected and weighed immediately after the rats were sacrificed, and the kidney index was calculated using the following formula: kidney index = kidney weight (g)/body weight (g).

### 2.4. Serological parameters

Assay kits were used to measure the contents of TG (Cat No. A110-1-1), TC (Cat No. A111-1-1), BUN (Cat No. C013-2-1), SA (Cat No. A028-2-1), and Scr (Cat No. C011-2-1), which were all purchased from Nanjing Jincheng Institute of Biotechnology (Jiangsu, China).

### 2.5. Histopathological examination

Kidney tissues were immersed in 4% paraformaldehyde for 24 h, embedded in paraffin, and cut into 5-μm thick sections. Hematoxylin and eosin (H&E) staining was performed following the manufacturer’s instructions (Beijing Leagene Biotech Co., Ltd., DH0006). Three different rats’ sections from each group.

### 2.6. Oil Red O staining

Oil Red O solution was prepared by dissolving 0.5 g of Oil Red O powder (Sigma, O0625) in 100 mL of isopropanol, and three parts of dissolved Oil Red O were mixed with two parts of water and then filtered. Renal tissues were frozen in OTC compound, cut into 7-μm thick sections, and fixed with 4% paraformaldehyde. The sections were rinsed with ddH_2_O for 5 min, rinsed with 60% isopropanol for 1 min, stained with Oil Red O solution for 30 min, and finally rinsed with 60% isopropanol. Nucleus were counterstained with hematoxylin. The stained sections were observed using an Olympus microscope. Three rats in each group were randomly selected for examination.

### 2.7. Western blot analysis

The appropriate amount of renal cortex was placed into homogenate tubes with an equal proportion of RIPA lysis buffer (Thermo Fisher) mixed with a protease inhibitor cocktail (Roche) and homogenized using a tissue grinder. Protein lysates were then prepared for western blot analysis. Proteins were isolated by electrophoresis on 12.5% SDS–PAGE gels, transferred onto PVDF membranes, and blocked with 5% non-fat milk for 2 h. The membranes were incubated overnight at 4 °C with the following primary antibodies: Cleaved caspase-3 (CST#9661, Cell Signaling Technologies), Bcl-2 (ab196495, Abcam), Bax (CST#2772, Cell Signaling Technologies), ADFP (ab108323, Abcam), CD36 (ab133625, Abcam), and GAPDH (CST#5174, Cell Signaling Technologies). The membranes were subsequently washed and then incubated for 60 min at room temperature with HRP-conjugated anti-mouse or anti-rabbit secondary antibodies. An ECL reagent kit (Millipore, USA) and gel imaging equipment (Bio-Rad, ChemiDocTM Touch, USA) were used to detect the presence of protein bands on the membrane. The quantification of the band intensities was performed using Image Lab 5.2.1 software (BIO-RAD), and the band intensities were normalized to GAPDH.

### 2.8. Quantification of GSH and MDA

The homogenates were centrifuged at 4500 rpm for 15 min at 4 °C, and the supernatants were taken to determine the levels of oxidative stress biomarkers, such as GSH (Cat No. A006-2-1) and MDA (Cat No. A003-1-2), using corresponding kits according to the manufacturer’s protocols (Nanjing Jiancheng Bioengineering Institute, Nanjing, China).

### 2.9. TUNEL and DAPI staining

Frozen kidney tissues were cut into 7-μm thick sections and fixed in 4% paraformaldehyde for 30 min. The sections were then incubated in PBS containing 0.4% Triton X-100 for 5 min followed by incubation in TUNEL solution (Beyotime Biotechnology, Shanghai, China, Cat No. C1090) for 1 h in a humidified chamber, and the sections were counterstained with DAPI (Solarbio, Cat No.C0065). The slides were sealed with an anti-fluorescence quencher and observed under a fluorescence microscope (Olympus, Japan), and images were acquired. Three experimental animals in each group were randomly selected for this experiment.

### 2.10. Statistical analysis

Graphing was performed with GraphPad Prism 9 and statistical analyses were performed using IBM SPSS 25 for Windows. Multiple group comparisons were performed using one-way ANOVA, and Tukey or Dunnett T3 methods were used for *post-hoc* analysis. Data are expressed as the mean ± *SD*, and *p*-values ≤ 0.05 indicated statistically significant differences.

## 3. Results

### 3.1. TGs protect kidneys against doxorubicin-mediated injury

**Figure 1.**
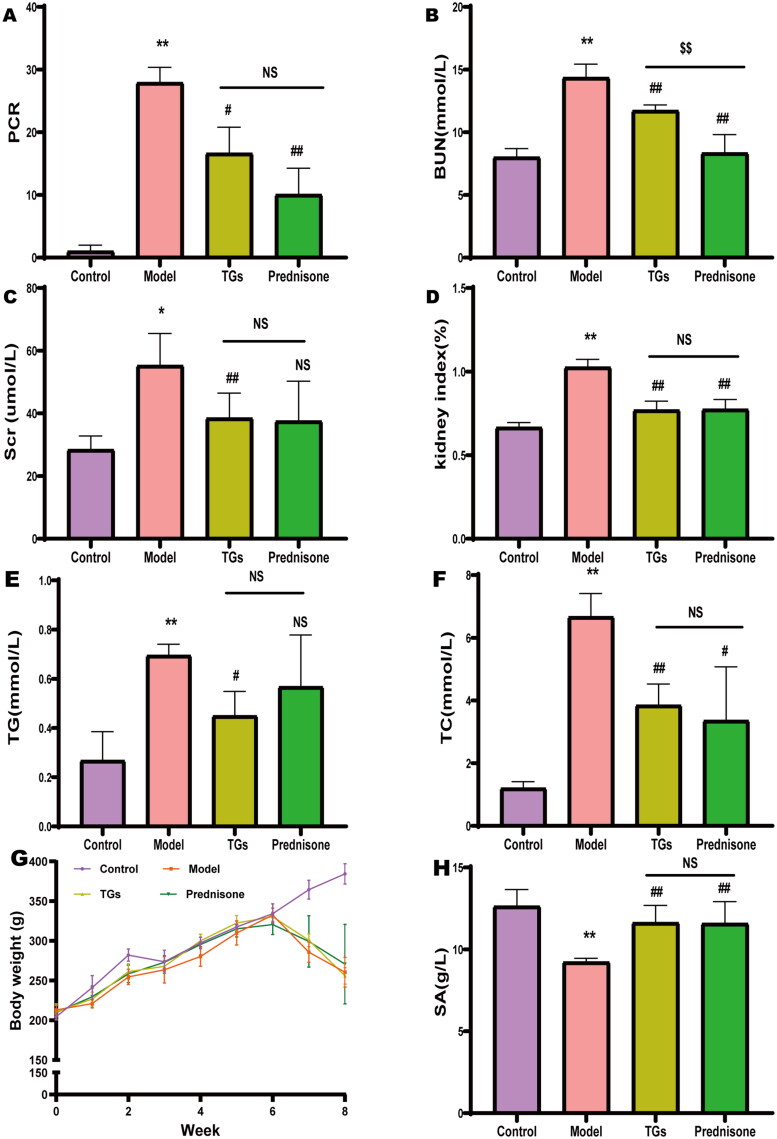
TGs protect kidneys against doxorubicin-mediated injury. (A) 24 h Urine protein-to-creatinine ratio (PCR). (B) Blood urea nitrogen. (C) Serum creatinine concentration. (D) Kidney index. (E) Serum TG levels in rats (*n* = 5–6). (F) Serum TC levels in rats (*n* = 5–6). (G) Body weight (*n*= 5–6 per group). (H) Serum albumin (*n*= 5–6 per group). Significance between groups was determined by ANOVA followed by Dunnett’s T3. Model group *vs.* Control group (*p ≤ 0.05 and **p ≤ 0.01); TGs group and Prednisone group *vs.* Model group (^#^*p* ≤ 0.05, ^##^*p* ≤ 0.01, and NS *p *> 0.05); TGs group *vs.* Prednisone group (^$$^*p* ≤ 0.01 and NS *p *> 0.05).

Rats were injected with DOX after 7–8 weeks, and PCR, BUN, Scr, kidney index, TG, and TC were significantly increased (Figures 1(A–F)), but significantly decreased body weight and SA (Figures 1(G,H)). These results demonstrated that the models were successful. In addition, TGs and prednisone ameliorated Dox-mediated renal injury, and the treatment effect of TGs was comparable to that of prednisone. However, TGs and prednisone did not reverse the body weight loss in rats. Compared to TGs, prednisone showed better effectiveness in reducing PCR and BUN.

### 3.2. TGs protect against alterations in the kidney architecture of NS

**Figure 2.**
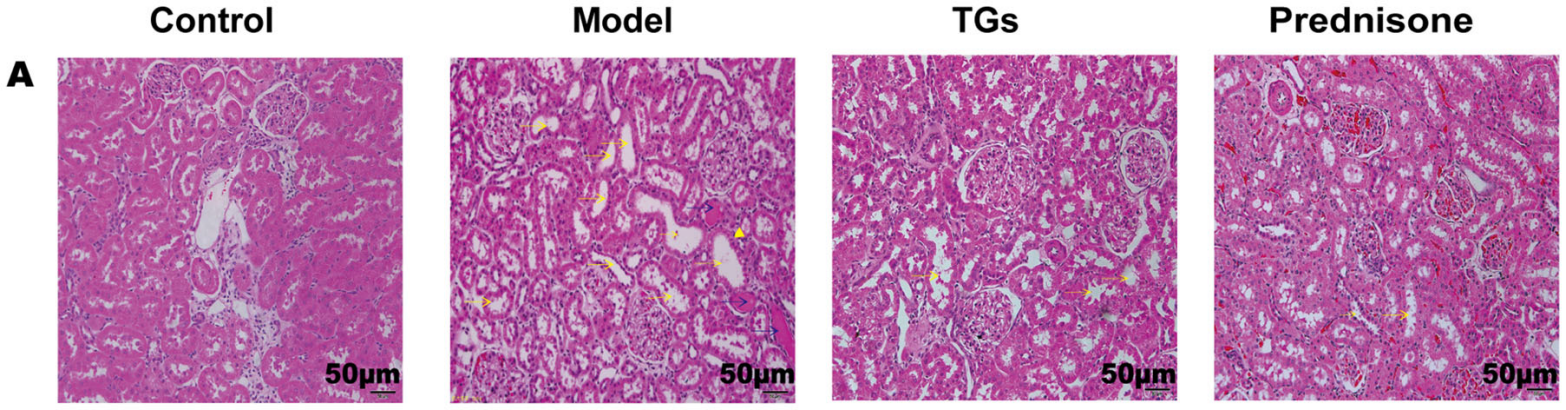
TGs protect against alterations in the kidney architecture of NS. HE staining (*n* = 3; scale bar: 50 µm). Yellow arrows indicate brush border detachment and absence. Blue arrows indicate protein casts. Yellow triangles indicate focal inflammatory cell infiltration.

The histopathological changes in the kidney tissues of rats in each group were observed by HE staining. The results showed that severe renal tubular damage occurred after DOX injection, including renal tubular dilation with granular degeneration and tubule brush border shedding as well as vacuolar degeneration of renal tubular epithelial cells, protein casts, and inflammatory cell infiltration (Figure 2). The therapeutic outcome in the TG group was equivalent to that in the prednisone group.

### 3.3. TGs alleviate abnormal lipids in NS

**Figure 3.**
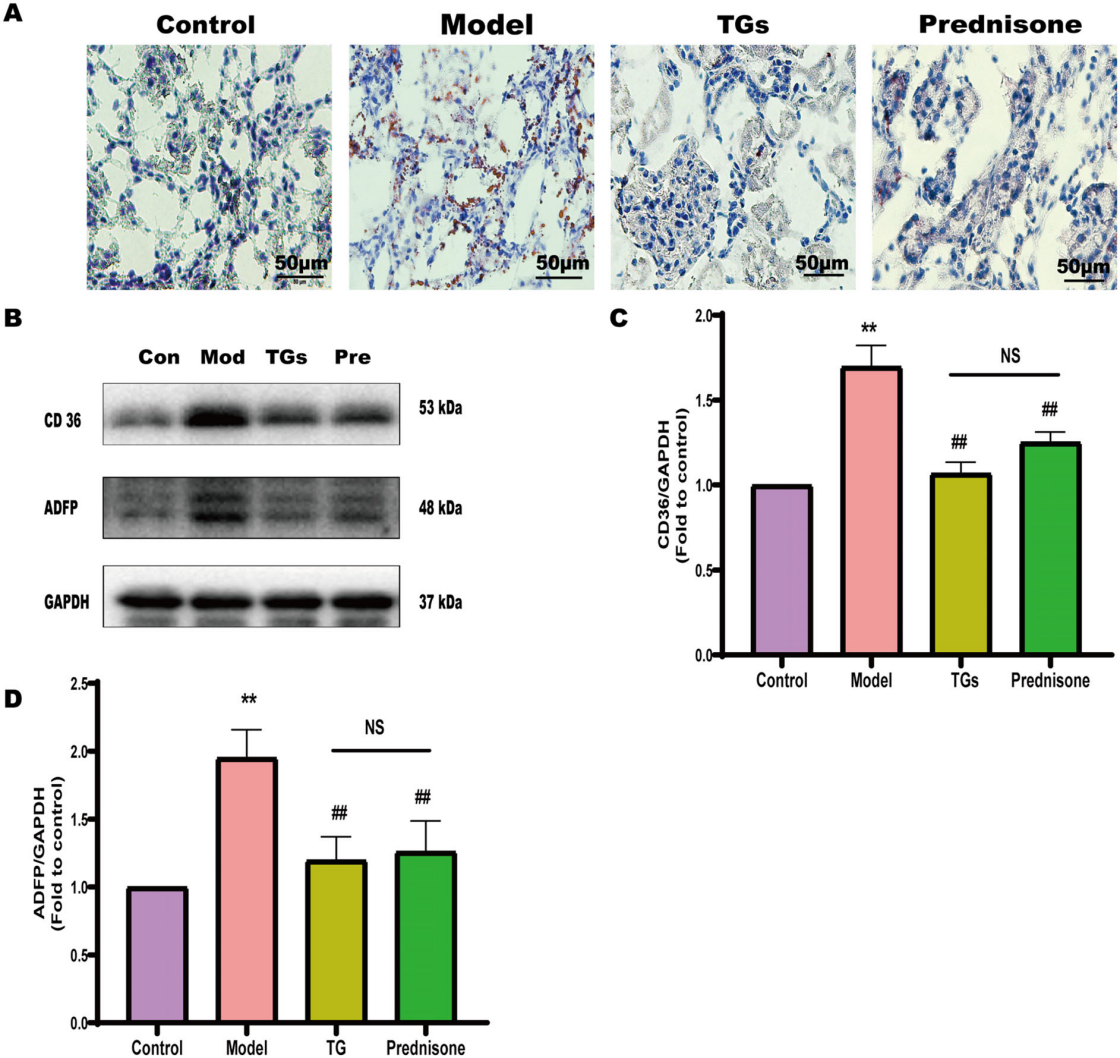
TGs alleviate abnormal lipids in NS. (A) Oil Red O and hematoxylin staining of kidney sections (*n* = 3; scale bar: 50 µm). (B–D) Expression of CD36 and ADFP in renal tissues (*n* = 3). Significance between groups was determined by ANOVA followed by Tukey. Model group *vs.* Control group (**p ≤ 0.01); TGs group or Prednisone group *vs.* Model group (^#^*p* ≤ 0.05 and ^##^*p* ≤ 0.01); TGs group *vs.* Prednisone group (NS *p *> 0.05).

To analyze the effect of TGs on lipid deposition in kidney tissue, Oil Red O staining was performed. Lipid droplets were not observed in normal rats. However, large orange-red lipid droplets were observed in the model group, and the orange-red droplets were mainly concentrated in renal tubules (Figure 3(A)). In addition, TGs reduced lipid deposition in the kidney tissue doxorubicin-induced NS rats. WB analysis also confirmed that TGs significantly decreased the expression of the CD36 and ADFP (two lipid-related proteins) (Figures 3(B–D)). Thus, these findings indicated that the therapeutic outcome of TG was better than that of prednisone.

### 3.4. TGs inhibit renal oxidative stress in NS

**Figure 4.**
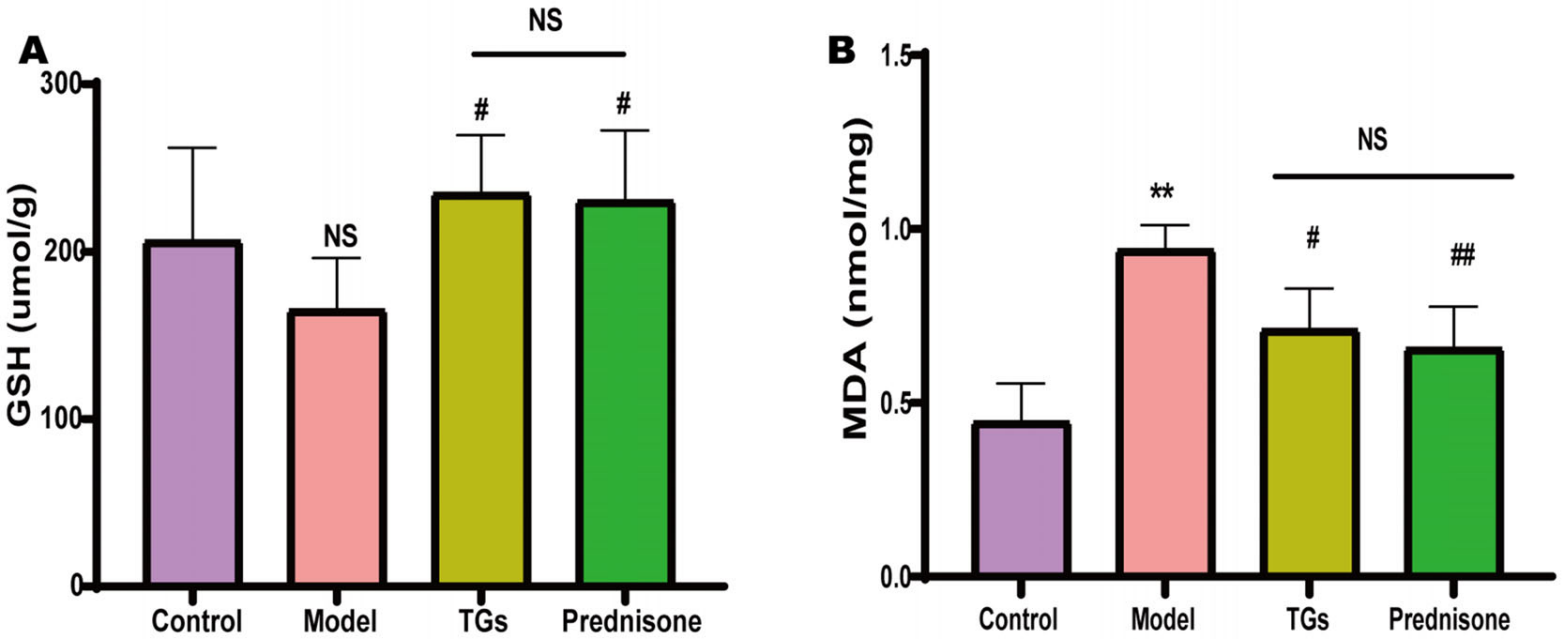
TGs inhibit renal oxidative stress in NS. (A) Levels of GSH in renal tissue homogenate. (B) Levels of MDA in renal tissue homogenate. (*n* = 5–6). Significance between groups was determined by ANOVA followed by Dunnett’s T3. Model group *vs.* Control group (**p ≤ 0.01, and NS *p *> 0.05); TGs group or Prednisone group *vs.* Model group (^#^*p* ≤ 0.05 and ^##^*p* ≤ 0.01); TGs group *vs.* prednisone group (NS *p *> 0.05).

Abnormal lipid metabolism induces lipid peroxidation, leading to oxidative stress. To evaluate whether TGs improve lipid-related oxidative stress, we quantified the amounts of GSH and MDA in rat kidney tissues. The results showed that DOX reduced GSH content (but not significantly) and increased MDA content in the kidneys. Treatment with TGs or prednisone restored the content of GSH and reduced the DOX-mediated increase in MDA (Figure 4). These findings indicated that TGs or prednisone significantly inhibit lipid peroxidation and restore antioxidant capacity to a certain extent in the kidneys of NS model rats. The TG therapeutic outcome was equivalent to that in the prednisone group.

### 3.5. TGs inhibit renal apoptosis of NS

**Figure 5.**
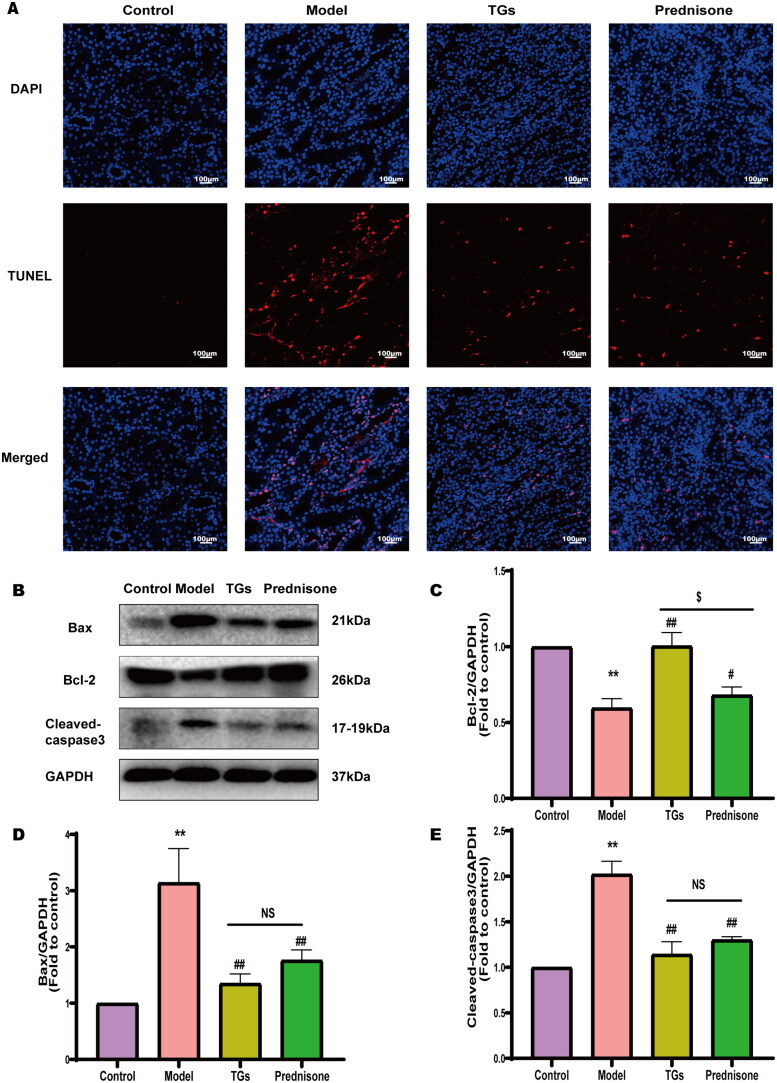
TGs inhibit renal apoptosis of NS. (A) TUNEL and DAPI staining of kidney sections (*n* = 3; scale bar: 100 µm). (B–E) Bax, Bcl-2, and Cleaved caspase-3 expression in the kidney (*n* = 3). Significance between groups was determined by ANOVA followed by Tukey. Model group *vs.* Control group (**p ≤ 0.01); TGs group or Prednisone group *vs.* Model group (^#^*p* ≤ 0.05 and ^##^*p* ≤ 0.01); TGs group *vs.* prednisone group (^$^*p* ≤ 0.05, and NS *p *> 0.05).

To determine the apoptosis levels of the kidneys, TUNEL assays and WB analyses were performed. TUNEL staining showed a significantly higher level of cell death in the model group than in the control group, TGs group and prednisone group significantly decreased the number of TUNEL-positive cells (Figure 5(A)). WB analysis indicated that the TGs and prednisone groups exhibited reduced levels of Bax and active Caspase-3 but increased levels of the Bcl-2 antiapoptotic protein compared to the model group (Figures 5(B–E)). The TGs therapeutic outcome was equivalent to that in the prednisone group.

## 4. Discussion

As early as 1982, the concept of lipid nephrotoxicity attracted much attention [11]. Hyperlipidemia is prevalent in NS and is considered to be a feature of severe NS [12]. Severe impairment of lipid clearance is a leading cause of abnormal lipid metabolism in NS, resulting in a surplus of fatty acids and glyceride converted to TG, which accumulate in the form of intracellular lipid droplets [13]. Non-adipose tissue has a limited capacity to store TGs [14], and excessive lipid deposition causes nephrotoxicity due to cellular dysfunction as well as accelerates energy metabolism, induces oxidative damage, and leads to cell death [15,16]. Therefore, reducing abnormal lipid deposition in the kidney mitigates kidney damage and slows the progression of NS. In the present study, DOX injection significantly increased the levels of PCR, BUN, Scr, TG, and TC but significantly decreased the levels of SA in rats compared to the model group, which indicated successful model construction. Treatment with TGs significantly reduced kidney damage in the NS model rats.

Oil Red O is a fat-soluble dye with can specifically bind to TG in tissues or cells to dye fat cells red [17]. As a scavenger receptor, CD36 is a key element in fatty acid uptake [18]. Overexpression of CD36 increases fatty acid uptake and directs abnormal lipid deposition and excessive oxidative stress [19,20]. CD36 is significantly upregulated in kidney disease and can reflect the severity of tissue injury in kidney disease to some extent [21]. Adipose differentiation-related protein (ADFP) is an important lipid droplet surface protein that is mainly involved in cellular fatty acid intake, lipid droplet formation, and lipid stores [22]. ADFP plays a role in preventing lipase entry into lipid droplets and slowing lipid digestion, allowing lipids to accumulate in lipid droplets [23]. Without ADFP, lipid droplets are degraded by the proteasome; thus, ADFP is an indicator of lipid accumulation [24,25].

In the present study, Oil Red O staining and western blot analysis indicated an accumulation of lipid droplets in the kidneys of model rats, but the amount of lipid droplets was significantly reduced after treatment with TGs. Moreover, the expression of CD36 and ADFP was also downregulated in the TGs group. These results demonstrated that TGs reduce abnormal lipid deposition in the kidneys of NS rats, thus improving renal injury.

Aberrant accumulation of lipids results in induced cellular oxidative stress [26]. As a representative product of lipid peroxidation, MDA directly reflects the extent of lipid peroxidation damage [27]. Because GSH is an important endogenous antioxidant in the body that scavenges oxidative free radicals and prevents a variety of diseases, it reflects the ability of the tissue to resist oxidative damage [28]. In the present study, the MDA content was significantly higher and the GSH level was significantly lower in the model group compared to the control group, which indicated that the kidneys of the model rats were in a state of lipid peroxidation. Moreover, treatment with TGs reduced the MDA content and increased the GSH level, thereby recovering the oxidative-antioxidative balance and reducing the cellular damage caused by lipid peroxidation.

Excessive lipid deposition may exceed the repair capacity of cells and lead to apoptosis [29]. Doxorubicin has a strong cytotoxic effect and induces cell apoptosis. Combining lipid deposition and doxorubicin may exacerbate injury at the same time. Bax is an important proapoptotic protein in the Bcl-2 family [30], and it is predominantly present in an inactive conformation, maintaining organismal stability in part through interaction with antiapoptotic Bcl-2 proteins; thus, Bax and Bcl-2 play a key role in cell death and survival [31]. Caspase 3 is a key member of the caspase family, and activation of Caspase 3 is an indispensable step in mitochondria-dependent apoptosis [32]. Its spliceosome, Cleaved caspase-3 is a key downstream factor in the apoptotic cascade and functions as an important executor of apoptosis [33]. In the present study, TGs prevented apoptosis in NS rats by decreasing the expression of Bax and Cleaved caspase-3 as well as restoring the expression of Bcl-2.

In summary, the present findings suggested that TGs treatment improves renal injury induced by doxorubicin by reducing renal lipid deposition, inhibiting renal lipid peroxidation, and mitigating cell apoptosis. However, the precise mechanism and signaling pathway through which TGs improve renal lipid deposition require additional detailed studies.

## Supplementary Material

Supplemental Material

## Data Availability

All data generated during the study can be obtained upon reasonable request from the corresponding author.
