# Supplementary figures and images for: Tripterygium glycosides improve abnormal lipid deposition in nephrotic syndrome rat models
Source: Ren Fail. 2023 Mar 6;45(1):2182617. doi: 10.1080/0886022X.2023.2182617 (PMC10013393; doi:10.1080/0886022X.2023.2182617)

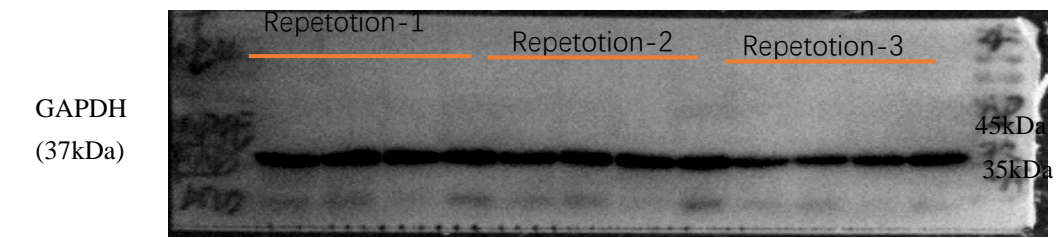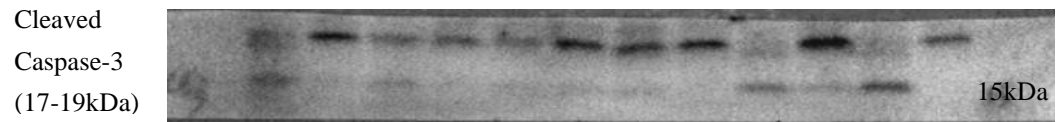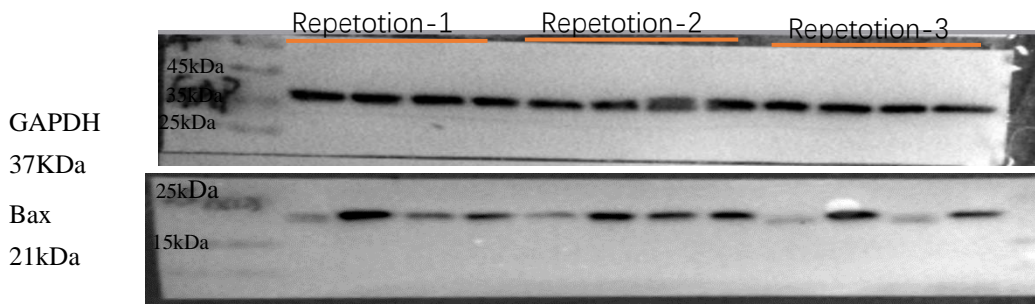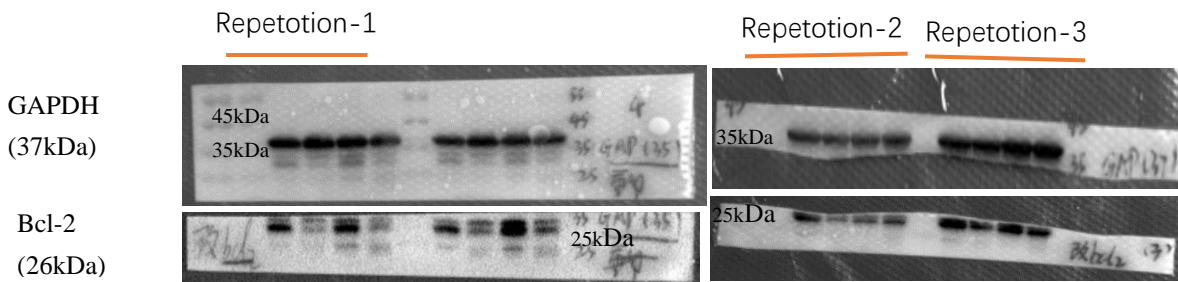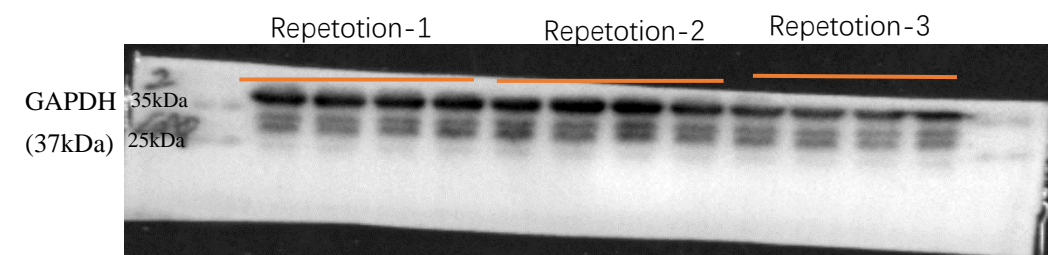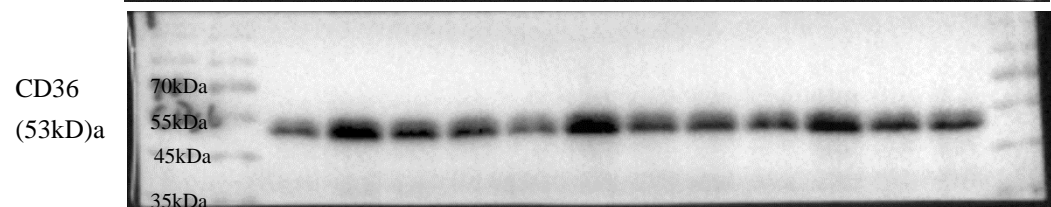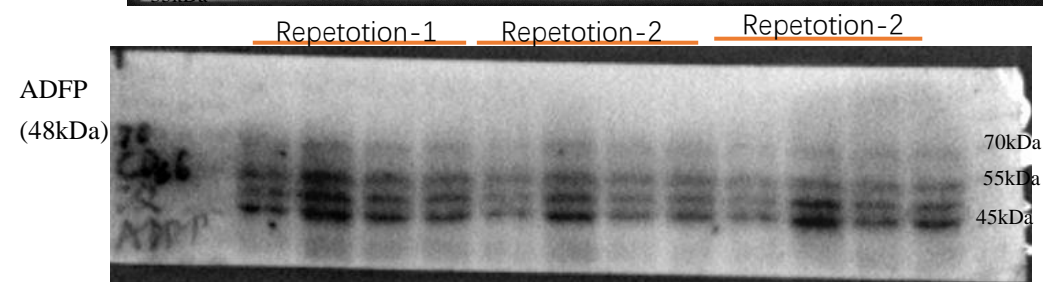

Supplement: Supplemental Material [file IRNF_A_2182617_SM8331.pdf]
